# Chronic heart failure with diabetes mellitus is characterized by a severe skeletal muscle pathology

**DOI:** 10.1002/jcsm.12515

**Published:** 2019-12-21

**Authors:** Jack O. Garnham, Lee D. Roberts, Ever Espino‐Gonzalez, Anna Whitehead, Peter P. Swoboda, Aaron Koshy, John Gierula, Maria F. Paton, Richard M. Cubbon, Mark T. Kearney, Stuart Egginton, T. Scott Bowen, Klaus K. Witte

**Affiliations:** ^1^ Leeds Institute of Cardiovascular and Metabolic Medicine University of Leeds Leeds UK; ^2^ School of Biomedical Sciences, Faculty of Biological Sciences University of Leeds Leeds UK

**Keywords:** HFrEF, Mitochondrial dysfunction, Atrophy, Exercise intolerance, Diabetes

## Abstract

**Background:**

Patients with coexistent chronic heart failure (CHF) and diabetes mellitus (DM) demonstrate greater exercise limitation and worse prognosis compared with CHF patients without DM, even when corrected for cardiac dysfunction. Understanding the origins of symptoms in this subgroup may facilitate development of targeted treatments. We therefore characterized the skeletal muscle phenotype and its relationship to exercise limitation in patients with diabetic heart failure (D‐HF).

**Methods:**

In one of the largest muscle sampling studies in a CHF population, *pectoralis major* biopsies were taken from age‐matched controls (*n* = 25), DM (*n* = 10), CHF (*n* = 52), and D‐HF (*n* = 28) patients. *In situ* mitochondrial function and reactive oxygen species, fibre morphology, capillarity, and gene expression analyses were performed and correlated to whole‐body exercise capacity.

**Results:**

Mitochondrial respiration, content, coupling efficiency, and intrinsic function were lower in D‐HF patients compared with other groups (*P* < 0.05). A unique mitochondrial complex I dysfunction was present in D‐HF patients only (*P* < 0.05), which strongly correlated to exercise capacity (*R* ^2^ = 0.64; *P* < 0.001). Mitochondrial impairments in D‐HF corresponded to higher levels of mitochondrial reactive oxygen species (*P* < 0.05) and lower gene expression of anti‐oxidative enzyme superoxide dismutase 2 (*P* < 0.05) and complex I subunit NDUFS1 (*P* < 0.05). D‐HF was also associated with severe fibre atrophy (*P* < 0.05) and reduced local fibre capillarity (*P* < 0.05).

**Conclusions:**

Patients with D‐HF develop a specific skeletal muscle pathology, characterized by mitochondrial impairments, fibre atrophy, and derangements in the capillary network that are linked to exercise intolerance. These novel preliminary data support skeletal muscle as a potential therapeutic target for treating patients with D‐HF.

## Introduction

Chronic heart failure (CHF) and type 2 diabetes mellitus (DM) remain two primary causes of mortality and morbidity.^1^ Approximately 25% of CHF patients also have coexistent DM [i.e. diabetic heart failure (D‐HF)], which is currently increasing in prevalence.^2^ Patients with D‐HF have worse symptoms, exercise limitation, and mortality compared with either CHF or DM.^3^, ^4^ However, the mechanisms underlying the pathophysiological phenotype of D‐HF patients, and therefore potential for targeted interventions, remain poorly established. This issue is further complicated by the finding that cardiac function is broadly similar between D‐HF and CHF patients,^5^ suggesting peripheral ‘non‐cardiac’ mechanisms substantially contribute to the adverse phenotype.

Patients with CHF develop significant skeletal muscle impairments, which impact exercise tolerance consequent to mitochondrial and metabolic derangements, fibre structural alterations, and impaired capillarity.^6^ The degree of these impairments, which are independent of cardiac function, are strongly correlated to exercise limitation and are thought to be underpinned by various mechanisms [e.g. reactive oxygen species (ROS), circulating inflammatory cytokines, and inactivity].^6^ To date, however, no study has extensively characterized the functional skeletal muscle phenotype in D‐HF and its association with exercise limitation when compared with CHF or DM. Such data would provide strong insight into whether a skeletal muscle pathology contributes to the pathogenesis of D‐HF and identify this as a specific therapeutic approach.

The present study, therefore, performed skeletal muscle biopsy sampling in humans with the aim of characterizing key indices (i.e. mitochondrial function, fibre structure, capillarity, and transcriptional regulators) between age‐matched controls (CON), CHF, DM, and D‐HF patients. We hypothesized that D‐HF would be associated with a distinctive muscle pathology that closely correlates to exercise limitation.

## Methods

### Participants

Clinical characteristics for all patients are presented in *Table* 1. Patients were grouped into four cohorts based upon their underlying condition, including CON, DM, heart failure (HF), and HF with diabetes mellitus (D‐HF). CON had no clinical evidence of CHF, a left ventricular ejection fraction (LVEF) >50% and no previous diagnosis of left ventricular systolic dysfunction. Patients in the DM group had a previous diagnosis (>3 months), as defined by a documented history of DM, fasting plasma glucose ≥7.0 mmol·L^−1^, plasma glucose ≥11.1 mmol·L^−1^ 2 h after the oral glucose tolerance test, and/or an HbA1c ≥6.5% (≥48 mmol·L^−1^). CHF patients had symptoms of stable CHF (>3 months on medical therapy), and a LVEF <50% as confirmed by echocardiography [following current European Society of Cardiology (ESC) guidelines].^7^ Patients with D‐HF met criteria for both CHF and DM groups as outlined earlier. All participants were indicated for device therapy with either a pacemaker, implantable cardioverter defibrillator, or cardiac resynchronization therapy device according to current indications.^7^ Patients with CHF and D‐HF performed a peak symptom‐limited exercise test to volitional exhaustion on a cycle ergometer for determination of pulmonary gas exchange (V̇O_2_ and V̇CO_2_) and ventilation (V̇_E_).^8^ Exclusion criteria included inability to provide informed consent due to cognitive dysfunction, or the presence of previous diagnoses with other potentially confounding comorbidities, such as other cardiovascular conditions, chronic obstructive pulmonary disease, or cancer. All patients provided written informed consent, and all procedures were conducted in accordance with the Declaration of Helsinki after receiving local institute ethical approval (11/YH/0291).

**Table 1:** Demographic, physical, and clinical characteristics of patients

	CON	DM	CHF	D‐HF
Participants [% (*n*)]	22 (25)	9 (10)	45 (52)	24 (28)
Male [% (*n*)]	64 (16)	−90 (9)	83 (43)	86 (24)
Age (years)	72.2 ± 2.0	74.5 ± 1.9	71.6 ± 1.6	71.4 ± 1.9
Weight (kg)	81.0 ± 3.4	105.6 ± 8.9**	81.0 ± 2.6†	88.6 ± 3.3‡
BMI	27.8 ± 1.1	33.3 ± 2.6	27.9 ± 0.8	28.8 ± 1.6
V̇O_2peak_ (mL·kg^−1^·min^−1^)			15.3 ± 0.9	13.0 ± 0.6
Clinical factors				
NYHA functional class [% (*n*)]				
I			7.7 (4)	3.6 (1)
II			55.8 (29)	50.0 (14)
III			36.5 (19)	46.4 (13)
Ischaemic aetiology [% (*n*)]			61.5 (32)	64.3 (18)
DCM aetiology [% (*n*)]			25.0 (13)	25.0 (7)
AF [% (*n*)]	48.0 (12)	30.0 (3)	17.3 (9)	28.6 (8)
CABG [% (*n*)]	28.0 (7)	10.0 (1)	21.2 (11)	25.0 (7)
Hypertension [% (*n*)]	36.0 (9)	60.0 (6)	32.7 (17)	57.1 (16)
LVEF (%)			24.8 ± 1.9	30.2 ± 2.2
LVIDd (mm)			58.5 ± 1.4	58.4 ± 1.9
Haemoglobin (g·L^−1^)	134.1 ± 3.7	140.8 ± 4.8	138.8 ± 2.2	128.1 ± 4.6
Sodium (mmol·L^−1^)	138.9 ± 0.8	136.3 ± 1.4	139.4 ± 0.7	132.8 ± 5.5
Potassium (mmol·L^−1^)	4.7 ± 0.1	4.6 ± 0.1	4.5 ± 0.1	4.6 ± 0.1
Creatinine (μmol·mL^−1^)	86.9 ± 4.0	106.7 ± 9.3	101.1 ± 5.9	106.3 ± 9.4
eGFR (mL·min^−1^·1.73 m^−2^)	69.4 ± 3.2	51.8 ± 5.5**	60.8 ± 2.6*	55.4 ± 3.6**
Plasma Glucose (mmol·L^−1^)		9.3 ± 1.9		8.1 ± 0.6
HbA1c (mmol·mol^−1^)		50.5 ± 6.9		62.3 ± 3.3

Data are expressed as mean ± SEM unless otherwise stated. AF, atrial fibrillation; CABG, coronary artery bypass graft; DCM, dilated cardiomyopathy; eGFR, estimated glomerular filtration rate; HbA1c, glycated haemoglobin; LVEF, left ventricular ejection fraction; LVIDd, left ventricular internal diameter at diastole; NYHA, New York Heart Association; V̇O_2_peak, peak pulmonary oxygen uptake.

* *P* < 0.05 vs. CON.

** P < 0.01 vs. CON.

† P < 0.05 vs. DM.

‡ P < 0.01 vs. DM.

### Muscle biopsy

Skeletal muscle biopsies of *pectoralis major* (~50 mg) were obtained from all participants during routine device implantation procedures. The biopsy was taken within 3 months following study recruitment and baseline clinical data collection. There were no complications or adverse events with this procedure. One piece of muscle sample was immediately placed in 1 mL of ice‐cold specialized preservation solution (BIOPS) for assessment of mitochondrial respiration,^9^ while two other portions were divided and rapidly frozen for subsequent histology and molecular analysis.

### Mitochondrial function

Mitochondrial respiration was assessed *in situ* from saponin‐permeabilized skeletal muscle fibres using high‐resolution respirometry (Oxygraph‐2K; Oroboros Instruments, Innsbruck, Austria).^9^ Briefly, (i) complex I leak respiration was determined by addition of glutamate (10 mM), malate (0.5 mM), and pyruvate (5 mM) (i.e. a measure of proton leak under non‐phosphorylating conditions); (ii) adenosine diphosphate (2.5 mM) was added to provide a measure of complex I oxidative phosphorylation (OXPHOS); (iii) outer mitochondrial membrane integrity was determined by addition of 10‐ μM cytochrome *c*; (iv) succinate at 10 mM as a complex II substrate provided complex I + II OXPHOS; (v) 5‐μM carbonyl cyanide 4‐(trifluoromethoxy)‐phenylhydrazone (FCCP) for maximal uncoupled complex I + II respiration; (vi) complex I inhibitor rotenone at 0.25 μM provided uncoupled complex II respiration; and (vii) 2.5‐μM antimycin A as a complex III inhibitor for residual oxygen consumption (ROX) to calculate non‐mitochondrial (background) respiration, which was then used to normalize the data. As an index of total mitochondrial ROS production, the Amplex UltraRed assay was used to measure H_2_O_2_ production in each respiratory state as previously described.^10^ Mitochondrial content was determined within the respirometer using a complex IV activity assay,^11^ by the addition of 0.5‐mM *N*,*N*,*N*',*N*'‐tetramethyl‐*p*‐phenylenediamine dihydrochloride (TMPD) as an artificial electron donor to complex IV in combination with 2‐mM ascorbate to maintain TMPD in a reduced state.^12^ Absolute mitochondrial respiration was normalized to complex IV activity to provide an index of mitochondrial intrinsic function. Respiratory control ratio (RCR; ratio of complex I OXPHOS to leak respiration) and flux control ratio (ratio of individual complex respiration to maximal uncoupled respiration) were also calculated.

### Immunohistochemistry

Immunohistochemistry methods have been described in detail previously.^13^ Briefly, in a subpopulation of patients, serial cross sections (10 μm thick) of frozen muscle biopsy samples were cut, mounted on to glass slides, and stained with primary antibodies against myosin heavy chain type I slow and type IIA fast oxidative (IgG2B, 1:1000; and IgG1, 1:500, respectively; Developmental Studies Hybridoma Bank, University of Iowa), with the remaining unstained fibres assumed as type IIx (fast glycolytic). Fibre boundaries were detected using an anti‐laminin antibody (1:200; L9393, Sigma‐Aldrich, St Louis, MO). Appropriate secondary antibodies were then applied. Capillaries were simultaneously stained with the marker for human endothelial cells, carbohydrate binding protein (lectin) biotinylated *Ulex europaeus* agglutinin I (1:200; B1065, Vector Labs, Peterborough, UK). Slides were imaged via an optical microscope (Nikon Eclipse E600, Nikon, Japan) attached to a digital camera (QIMAGING, MicroPublisher™ 5.0 RTV, Surrey, BC, Canada) and analysed using digital image software (AcQuis, Syncroscopy, Cambridge, UK). Fibre cross‐sectional area (FCSA), capillary‐to‐fibre ratio (C:F; # of capillaries to # of fibres), capillary density (CD; # of capillaries per tissue area), fibre‐type specific measures of local C:F (LCFR), and capillary density (LCD) were determined alongside heterogeneity of capillary distribution (i.e. logarithmic standard deviation of capillary domain area), using the automated software package DTect as described in extensive detail elsewhere.^13^ The C:F and CD offer a global perspective of muscle capillarity, while LCFR and LCD provide insight at the level of individual fibres. As capillarity is sensitive to changes in FCSA, LCD is particularly useful in assessing the influence of fibre atrophy.^13^

### Gene expression

RNA was extracted and purified from snap‐frozen muscle tissue using the RNeasy® Fibrous Tissue Mini Kit (Qiagen, Hilden, Germany). RNA concentrations (ng·μL^−1^) were quantified and reverse transcribed to cDNA; mRNA expression was determined using real‐time quantitative PCR with SYBR® Green ROX™ qPCR Mastermix (QIAGEN, Hilden, Germany) and a qPCR system (Applied Biosystems Prism 7900HT, Foster City, CA). Primers of key regulators of mitochondrial morphology were purchased from Qiagen including peroxisome proliferator‐activated receptor γ coactivator‐1α, superoxide dismutase 2 (SOD2), mitochondrial fission 1 (FIS1), optic atrophy 1 (OPA1), NADH:ubiquinone oxidoreductase core subunit S1 (NDUFS1), and NADH:ubiquinone oxidoreductase core subunit S3 (NDUFS3). Expression levels were normalized to an endogenous control, beta‐actin (ACTB), using the Δ‐Δ‐C_T_ method^14^, and then expressed relative to CON.

### Statistical analysis

Outliers were identified and removed using the regression and outlier removal method, as previously described.^15^ Assumption of homogeneity of variance was confirmed using Levene's test, while Shapiro–Wilk and Kolmogorov–Smirnov normality tests confirmed normal (Gaussian) distribution. Continuous variables were compared between cohorts using one‐way (one factor) 1 × 4 analysis of variance, with post hoc analyses performed using Tukey's multiple comparisons test if significance was detected. Unpaired Student's *t*‐tests was used to compare two cohorts where appropriate. Categorical variables (e.g. clinical variables) were compared using χ^2^ testing (or Fisher's exact test where appropriate). Pearson correlations examined relationships between continuous variables. Data are expressed as mean ± standard error of the mean unless otherwise stated. Statistical significance was accepted as *P* < 0.05.

## Results

### Patients

The demographic and physical characteristics of the 115 included patients are presented in *Table* 1, with pharmacological and device therapies presented in *Table* 2. Patients in the CHF and D‐HF groups had no differences in terms of cardiac function, disease severity (i.e. NYHA), VO_2peak_, or aetiology, drug/device therapies (except furosemide dose). Furthermore, no differences were present in terms of glycaemic measures between the DM and D‐HF patients (i.e. plasma glucose or HbAc1).

**Table 2:** Pharmacological treatments and device therapy

	CON	DM	CHF	D‐HF
Pharmacological treatments				
ACEi use [% (*n*)]	36.0 (9)	50.0 (5)	61.5 (32)	53.6 (15)
Ramipril equivalent dose (mg)	4.3 ± 1.1	8.3 ± 1.7	6.8 ± 0.9	7.0 ± 0.8
Beta‐blocker use [% (*n*)]	32.0 (8)	60.0 (6)	86.5 (45)^**†^	85.7 (24)^**†^
Bisoprolol equivalent dose (mg)	3.0 ± 0.6	2.9 ± 0.6	5.2 ± 0.5^*†^	7.6 ± 0.7
Loop diuretic use [% (*n*)]	16.0 (4)	30.0 (3)	48.1 (25)*	64.3 (18)^**†^
Furosemide equivalent dose (mg)	55 ± 15	33 ± 7	45 ± 4	100 ± 20^†¶^
ARB use [% (*n*)]			21.2 (11)	32.1 (9)
Candesartan equivalent dose (mg)			15.3 ± 4.2	16.6 ± 3.1
Aldosterone antagonist use [% (*n*)]			44.2 (23)	57.1 (16)
Aldosterone antagonist dose (mg)			26.1 ± 1.1	1 29.7 ± 5.1
Statin use [% (*n*)]	48.0 (12)	90.0 (9)	63.5 (33)	67.9 (19)
Statin dose (mg)	35.0 ± 6.9	38.8 ± 7.2	46.4 ± 4.1	44.2 ± 6.2
Aspirin use [% (*n*)]	20.0 (5)	10.0 (1)	46.2 (24)^*†^	46.4 (13)^*†^
Metformin use [% (*n*)]		50.0 (5)		46.4 (13)
Insulin use [% (*n*)]		20.0 (2)		10.7 (3)
Device therapy				
PPM [% (*n*)]	96.0 (24)	90.0 (9)		
ICD [% (*n*)]	4.0 (1)	10.0 (1)	26.9 (14)	3.6 (1)
CRT [% (*n*)]			73.1 (38)	96.4 (27)

Data are expressed as mean ± SEM unless otherwise stated. ACEi, angiotensin‐converting enzyme inhibitor; ARB, angiotensin receptor blocker; CRT, cardiac resynchronization therapy; ICD, implantable cardioverter defibrillator; PPM, permanent pacemaker.

* *P* < 0.05 vs. CON.

** *P* < 0.01 vs. CON.

† *P* < 0.05 vs. DM.

‡ P < 0.01 vs. DM.

¶ P < 0.5 vs. CHF.

### Mitochondrial function

**Figure 1:**
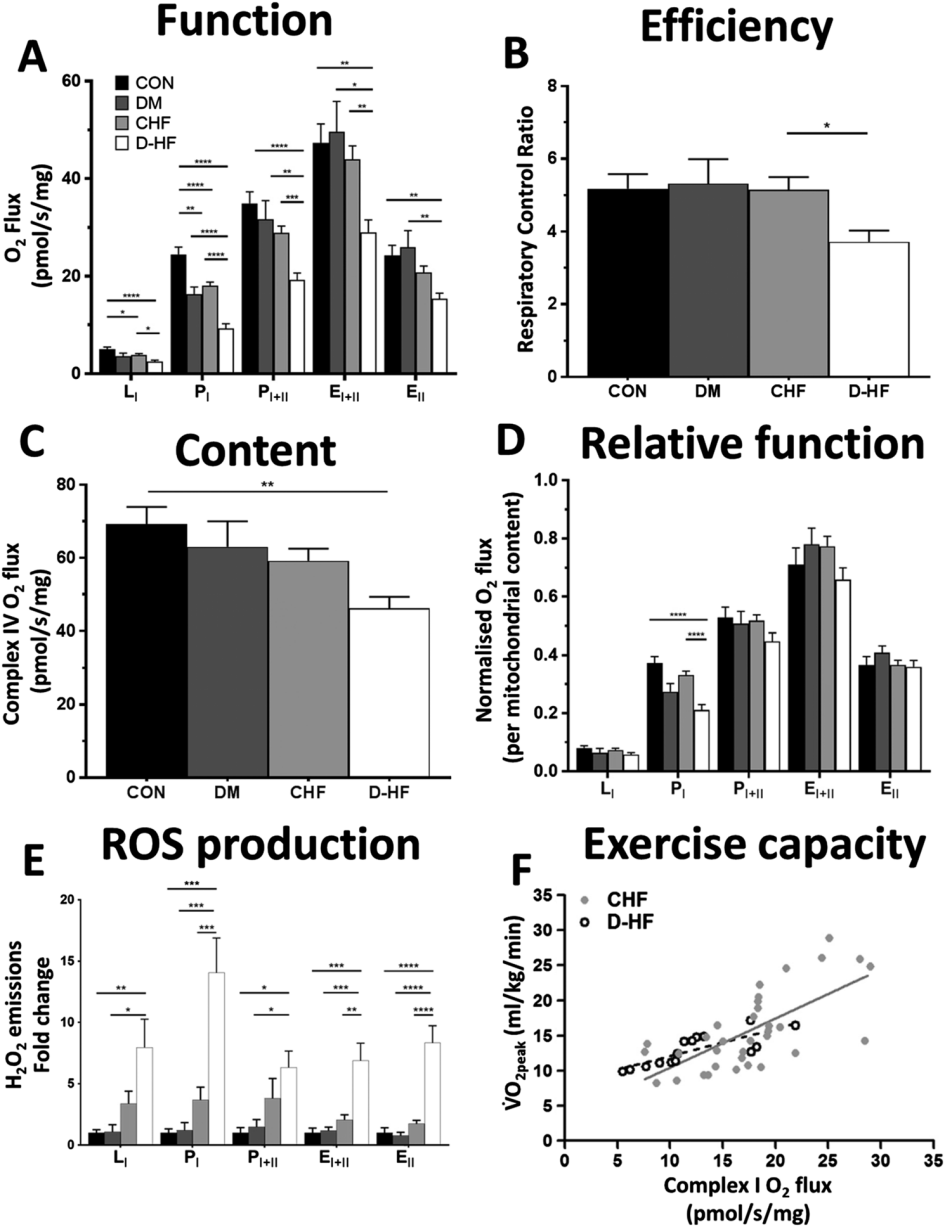
Mitochondrial function is impaired in the skeletal muscle of patients with D‐HF. Oxygen flux in all respiratory states (A) and the mitochondrial coupling efficiency as indicated by the respiratory control ratio (RCR) (B) is lower in D‐HF patients compared with DM and CHF. Mitochondrial content (measured by complex IV activity) is the lowest in D‐HF patients (C), and impairments at complex I remain despite normalizing for the lower mitochondrial content (D). These impairments corresponded to higher concentrations of mitochondrialderived reactive oxygen species (ROS) across all respiratory states in patients with D‐HF (E). N = 25, 10, 52, and 28 for CON, DM, CHF, and D‐HF, respectively. *P < 0.05; **P < 0.01; ***P < 0.001; ****P < 0.0001. Complex I function was strongly correlated to VO_2peak_ as a measure of whole‐body exercise capacity in both patients with CHF (R^2^ = 0.47; P < 0.001; solid line; N = 34) and even more so in D‐HF (R^2^ = 0.64; P < 0.001; dashed line; N = 15) (F). E_I + II_, maximal uncoupled complex I + II respiration; E_II_, uncoupled complex II respiration; L_I_, complex I leak respiration; P_I_, complex I oxidative phosphorylation; P_I + II_, complex I + II oxidative phosphorylation.

Mean group O_2_ flux for each mitochondrial respiratory state are presented in *Figure* 1A. Differences were detected between groups for each respiratory state, with complex I OXPHOS in D‐HF 43% (*P* = 0.008; *Figure* 1A) and 49% (*P* < 0.0001; *Figure* 1A) lower than DM and CHF, respectively. For complex I + II OXPHOS, differences were seen in D‐HF (*P* < 0.0001 vs. CON; *P* = 0.007 vs. DM; *P* = 0.001 vs. CHF; *Figure* 1A). This same trend was seen for uncoupled complex I + II respiration with D‐HF being lower than each respective cohort (*P* = 0.003 vs. CON; *P* = 0.016 vs. DM; *P* = 0.004 vs. CHF; *Figure* 1A). For complex II‐supported ETS capacity, D‐HF was lower than both CON (*P* = 0.003) and DM (*P* = 0.011; *Figure* 1A). The RCR (i.e. an index of mitochondrial coupling efficiency) was 28% lower in D‐HF compared with CHF (*Figure* 1B), with similar trends compared with CON (*P* = 0.070) and DM (*P* = 0.175). The O_2_ flux measured at complex IV (i.e. a proxy for mitochondrial content) is presented in *Figure* 1C. Differences were detected, with post hoc tests revealing that O_2_ flux was 33% lower in D‐HF than in CON (*P* = 0.001; *Figure* 1C).

**Figure 2:**
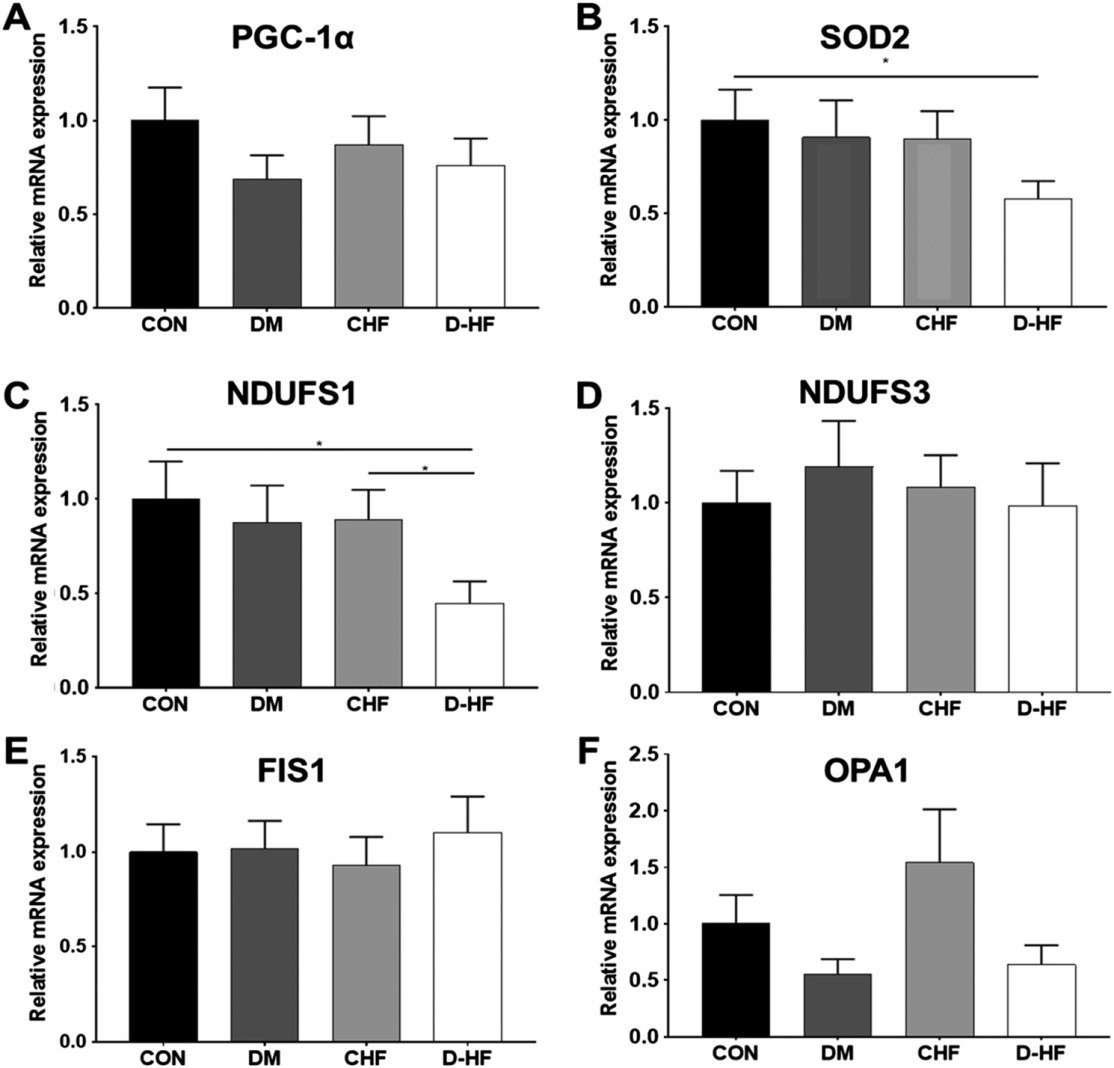
Gene expression of key mitochondrial‐regulating proteins in skeletal muscle across all patient groups, including (A) peroxisome proliferator‐activated receptor γ co‐activator 1α (PGC1α), (B) superoxide dismutase 2 (SOD2), (C) NADH:ubiquinone oxidoreductase core subunit S1 (NDUFS1), (D) NADH:ubiquinone oxidoreductase core subunit S3 (NDUFS3), (E) mitochondrial fission 1 protein (Fis1), (F) mitochondrial dynamin‐like GTPase/optic atrophy 1 (OPA1). N = 13–20 per group. *P < 0.05.

To account for differences in mitochondrial content, the absolute O_2_ flux measures for each respiratory state were normalized to complex IV activity (*Figure* 1D), which revealed complex I OXPHOS to be the only respiratory state that remained significantly different between the four cohorts (*P* < 0.0001), where D‐HF was 43% and 36% lower than CON and CHF, respectively (*P* < 0.0001; *Figure* 1D). Mitochondrial dysfunction is closely linked with increased oxidative stress. We therefore measured mitochondrial‐derived ROS generation and found this to be substantially higher in D‐HF (*Figure* 1E). Most notably, during complex I OXPHOS (P_I_), H_2_O_2_ production was ~14‐fold, ~12‐fold, and ~4‐fold higher in D‐HF compared with CON (*P* < 0.0001), DM (*P* = 0.0001), and CHF (*P* = 0.0009), respectively (*Figure* 1E). To examine the relationship between the skeletal muscle metabolic phenotype and our clinical data, *in situ* mitochondrial function was correlated to *in vivo* measures of exercise capacity in CHF and D‐HF patients, with complex I OXPHOS more strongly correlated to V̇O_2peak_ in D‐HF (*R* ^2^ = 0.64; *P* < 0.001) compared with CHF (*R* ^2^ = 0.47; *P* < 0.001) (*Figure* 1F). We next probed for potential underlying molecular regulators of mitochondrial dysfunction in D‐HF (*Figure* 2), which revealed that the expression of two key genes was down‐regulated in patients with D‐HF. This included the mitochondrial‐located antioxidant SOD2 (*Figure* 2B) and the key respiratory complex I subunit NADH:ubiquinone oxidoreductase core subunit S1 (NDUFS1) (*Figure* 2C). Overall, these data confirm that patients with D‐HF have impairments in skeletal muscle mitochondrial regulation that are correlated to exercise limitations.

### Fibre morphology

**Figure 3:**
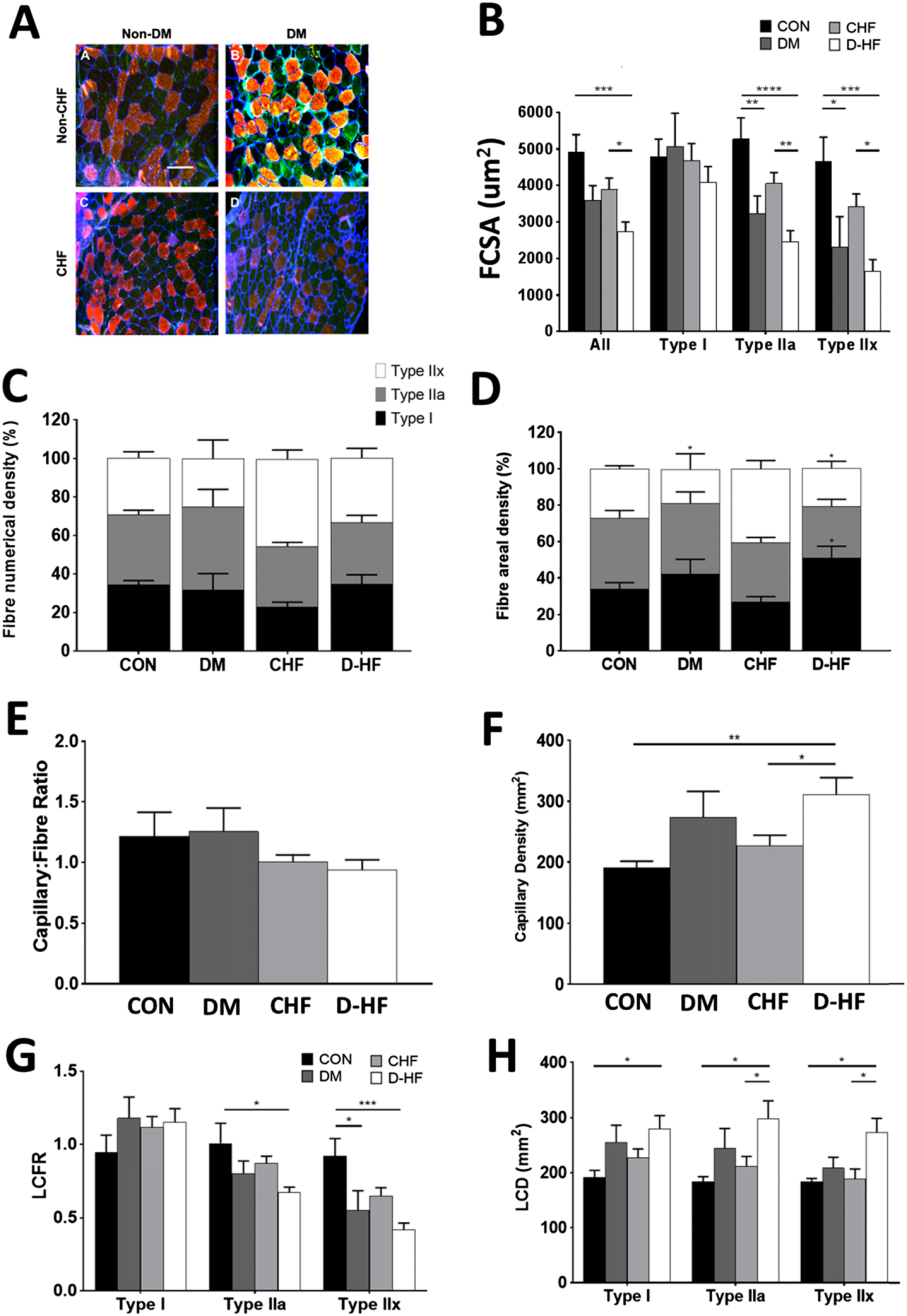
Representative composite images of stained muscle sections from the CON, DM, CHF, and D‐HF patient groups (A). Type I MHC fibres are stained red; type IIA MHC fibres are stained green; type IIX unstained/black; and the basal membrane is stained blue. Scale bar represents 200 μm. Mean fibre cross‐sectional area (FCSA) across each cohort according to fibre type shows greater atrophy in patients with D‐HF (B), with fibre‐type proportion (C) also presented. For fibre areal density (D), CHF resulted in a higher type IIX compared with DM or D‐HF. Global (E, F) and localized (G, H) indices of capillarization were also assessed, with D‐HF associated with a lower local capillary: fibre ratio (LCFR; G) yet higher global capillary density (CD; F) and local CD (LCD; H). N = 5–8 per group. *P < 0.05; **P < 0.01; ***P < 0.001; ****P < 0.0001.

We next investigated whether patients with D‐HF presented with gross deficits in fibre structure and composition. *Figure* 3A shows representative muscle sections from patients. Mean FCSA in D‐HF patients was 44% and 30% smaller compared with CON (*P* = 0.001) and CHF (*P* = 0.044), respectively (*Figure* 3B). In particular, fibre atrophy was type II specific, with type IIa FCSA 53% and 39% smaller in D‐HF compared with CON (*P* < 0.0001) and CHF (*P* = 0.001), respectively (*Figure* 3B), and type IIx FCSA was 65% and 52% smaller in D‐HF compared with CON (*P* = 0.0003) and CHF (*P* = 0.014), respectively (*Figure* 3B). Type IIa and IIx FCSA was also smaller in DM compared with CON (*P* < 0.01; *Figure* 3B), with a similar trend also seen in CHF (*P* = 0.115). No significant differences were found between D‐HF and DM groups, despite fibres in D‐HF showing a consistent trend to be smaller across all fibre types (*Figure* 3B). No muscle fibre type shifts were observed among groups; however, CHF had a higher areal density of type IIx fibres compared with DM (*P* = 0.040) and D‐HF (*P* = 0.035), respectively (*Figure* 3D). D‐HF also had a higher areal density of type I fibres compared with CHF (*P* = 0.014; *Figure* 3D).

### Fibre capillarity

No differences in the global C:F existed between groups (*Figure* 3E); however, fibre‐type specific C:F (i.e. LCFR) was lower in D‐HF patients by 34% in type IIa fibres (*P* = 0.011; *Figure* 3G) and 55% (*P* = 0.0003) in IIx fibres compared with CON. Additionally, the LCFR was lower in DM compared with CON for type IIx fibres (*P* = 0.023; *Figure* 3G). In contrast, a global index of CD was 63% and 37% higher in D‐HF compared with CON (*P* = 0.007) and CHF (*P* = 0.034), respectively (*Figure* 3F). Fibre‐type specific CD (i.e. LCD) was also higher in D‐HF compared with CON across all fibre types (*Figure* 3H), and also 41% (*P* = 0.0499) and 45% (*P* = 0.0174) higher in D‐HF compared with CHF for type IIa and type IIx fibres, respectively (*Figure* 3H). However, there were no differences in the spatial heterogeneity of capillary distribution between cohorts, with logarithmic standard deviation averaging 0.16 ± 0.01 for all groups.

## Discussion

This study has confirmed that patients with D‐HF demonstrate a severe skeletal muscle phenotype, manifesting as distinct mitochondrial impairments, fibre atrophy, and capillary remodelling. Notably, our data revealed an intrinsic mitochondrial complex I dysfunction specific to D‐HF, which corresponded to higher levels of mitochondrial ROS production and lower gene transcripts of complex I. Clinically, skeletal muscle mitochondrial impairments were closely correlated to whole‐body exercise limitations, providing potential evidence of a novel therapeutic target in the treatment of patients with D‐HF.

### Mitochondrial dysfunction in heart failure and diabetes

Many studies have investigated skeletal muscle mitochondrial function in either CHF or DM patients; however, the combined impact in D‐HF remains poorly investigated. This may have important implications given that D‐HF patients have poorer outcomes compared with either CHF or DM alone in terms of exercise intolerance, quality of life, and mortality.^4^, ^5^, ^16^, ^17^, ^18^ A lower mitochondrial content (rather than intrinsic function) is most often reported in patients with either CHF^19^, ^20^, ^21^ or DM^22^, ^23^, ^24^ compared with matched CON. Here, we show for the first time that patients with D‐HF have decrements in not only mitochondrial content but also functional impairments that particularly resided at the complex I. Interestingly, we demonstrated that absolute mitochondrial O_2_ flux is lower in both CHF and DM patients compared with CON, and this effect was exacerbated in patients with D‐HF. Importantly, we showed the depressed mitochondrial respiration could be largely explained by a lower mitochondrial content in patients with CHF or DM (following appropriate normalization). In contrast, however, D‐HF patients still presented with lower complex I respiration (i.e. intrinsic dysfunction) compared with other groups, even after correcting for mitochondrial content. This was confirmed by the mitochondrial coupling efficiency ratio (i.e. the RCR) also being impaired in the D‐HF group only. Thus, while the lower values of mitochondrial oxygen flux fits with the common observation of reduced mitochondrial content in CHF^19^, ^20^, ^21^ or DM^22^, ^23^, ^24^, the lower intrinsic flux through complex I in D‐HF highlights a greater problem in this specific cohort of patients that goes beyond simply a mitochondrial content issue. Moreover, our data show that the complex I specific impairment in the D‐HF patients is strongly correlated to exercise capacity (i.e. V̇O_2peak_), linking the pathophysiology of whole‐body exercise intolerance to skeletal muscle mitochondrial dysfunction in D‐HF and suggesting this could be a potential therapeutic target. Of note, these indices were more strongly correlated in patients with D‐HF compared with CHF alone (*Figure* 1F), suggesting complex I dysfunction may provide a mechanism to explain why V̇O_2peak_ is lower in D‐HF compared with CHF patients, likely by impairing muscle O_2_ extraction.

The underlying factor(s) driving the mitochondrial complex I dysfunction in D‐HF remains unclear. However, our data implicate a potential role for mitochondrial‐derived ROS. Mitochondrial ROS production was substantially elevated and proportionally greater at complex I in patients with D‐HF compared with other groups, while the gene expression of the mitochondrial antioxidant SOD2 was lower which may have compromised ROS clearance. Regarding the latter, recent data have shown levels of SOD2 are able to modulate mitochondrial ROS and dysfunction alongside atrophy in an animal model of mechanical ventilation.^25^ Taken together, the current data indicate a shift towards a pro‐oxidant state in D‐HF^26^ consistent with the observation that treatment with antioxidant therapies such as mitochondrial‐specific drugs or whole‐body exercise training can ameliorate oxidative stress in skeletal muscle and improve mitochondrial function, insulin sensitivity, and exercise capacity in CHF^27^, ^28^, ^29^ and DM.^30^, ^31^ A further reason may also be related to changes in the quality of mitochondrial complex I.^32^ NDUFS1 is the largest (75 kDa) subunit of complex I and forms a component of the eight iron‐sulphur chains involved in transferring electrons from NADH oxidation at the flavin mononucleotide to the ubiquinone binding site where the electron acceptor, ubiquinone, is reduced to ubiquinol.^33^ We found transcription of NDUFS1 was down‐regulated in D‐HF, and this may help explain, at least in part, a mechanism for impaired mitochondrial complex I respiration. Additional factors that may have caused the mitochondrial dysfunction also include insulin resistance, chronic hyperglycaemia, elevated inflammatory cytokines, substrate overload, and accumulation of intracellular lipids, although further studies are required to establish their contribution.

### Fibre atrophy and capillarity

Loss of muscle mass is common in many diseases and limits both exercise tolerance and quality of life.^6^ Patients with D‐HF demonstrated a severe muscle fibre atrophy, which tended to be greater when compared with the other groups. The mechanisms mediating greater fibre atrophy in D‐HF remains unclear; however, elevated mitochondrial ROS has been shown to directly induce atrophy and treatment with mitochondrial‐specific antioxidants can prevent this, at least in animal models.^34^ Thus, a higher production of mitochondrial ROS in D‐HF may drive the greater fibre atrophy observed. Our findings also identified that D‐HF causes a preferential atrophy of type II fibres, with type I fibres largely unaffected, which are in line with the type II isoform being more susceptible to atrophy across various chronic diseases.^35^

To assess potential O_2_ delivery limitations in D‐HF, the current study also quantified skeletal muscle capillarity, using both global and local measures of capillarity, which allowed us to gain greater insight compared with the standard approach.^13^, ^36^ Consistent with allometric scaling, we observed an increase in capillary density (CD) in D‐HF patients, which was matched by comparable increases in the local fibre‐type specific CD measures, that is, explained by the severe fibre atrophy seen in D‐HF compared with the other groups. This could even be interpreted as a shift towards improved capillary supply in D‐HF, for example, as part of a compensatory response to maintain O_2_ delivery. In contrast, when we applied the scale‐independent measure of local C:F (i.e. LCFR),^13^ we were able to detect fibre‐type specific reductions in potential O_2_/substrate supply from capillaries surrounding both type IIa and IIx fibres to be greatest in D‐HF patients. It is interesting to note that these differences were not evident when the global measure of C:F was quantified, highlighting the increased sensitivity of the LCFR method when muscle atrophy is present.^13^ Previous studies looking at global capillarity in CHF or DM have produced variable conclusions, with reports of either an increase^37^, ^38^ or decrease^39^, ^40^, ^41^, with such disparities likely explained by differences in the evaluation methods employed, disease severity, and/or degree of fibre atrophy. Another possibility is that capillary rarefaction may have preceded fibre atrophy^42^, such that earlier impairments to the capillary network in D‐HF went undetected. Overall, therefore, the current data suggest alterations to both microvascular O_2_ transport (indicated by our capillary measures) in addition to O_2_ utilization (indicated by our mitochondrial measures) may combine with a reduced muscle mass to exacerbate whole‐body exercise intolerance in patients with D‐HF. Of note, our data indicate that fibre atrophy was proportionally greater than the capillary rarefaction in patients with D‐HF (while proportional in CHF), which seems to further reinforce greater impairments in the maintenance of muscle mass (due, e.g. to insulin resistance) occur in the combination of both CHF and DM.

### Limitations

This study was limited by the observational design, which allowed characterization of variables and their relationships rather than prove causality. Further mechanistic studies are therefore warranted. All patients were referred for device implantation and whether our findings apply to the wider population is unknown. We also did not have a measure of exercise intolerance (i.e. V̇O_2peak_) in CON and DM groups to evaluate their current levels of aerobic fitness, while we cannot comment on whether current levels of physical activity may have influenced our results.^20^ However, detraining does not fully explain mitochondrial deficits in HFrEF^43^, and our data were from the *pectoralis major* which is likely not impacted to the same degree as the lower limbs by detraining. However, a further study comparing upper vs. lower limbs muscle alterations (e.g. *pectoralis major* and *vastus lateralis*) in the current patient groups would be worthy of future investigation.

## Conclusions

This study provides novel evidence that patients with D‐HF suffer severe skeletal muscle mitochondrial dysfunction, fibre atrophy, and capillary remodelling when compared with CHF or DM patients. The mitochondrial dysfunction is the result of both qualitative and quantitative alterations at complex I, which was well correlated to exercise intolerance and was paralleled by elevated mitochondrial‐derived ROS and impaired mitochondrial gene transcription. Targeting the skeletal muscle may therefore represent a novel therapy for the treatment of exercise intolerance in patients with D‐HF.

## Conflict of Interest

None declared.
